# Genes associated with inflammation may serve as biomarkers for the diagnosis of coronary artery disease and ischaemic stroke

**DOI:** 10.1186/s12944-020-01217-7

**Published:** 2020-03-12

**Authors:** Peng-Fei Zheng, Fu-Jun Liao, Rui-Xing Yin, Lu-Zhu Chen, Hui Li, Rong-Jun Nie, Yong Wang, Pei-Juan Liao

**Affiliations:** 1 Department of Cardiology, Institute of Cardiovascular Diseases, the First Affiliated Hospital, Guangxi Medical University, 6 Shuangyong Road, Nanning, 530021 Guangxi People’s Republic of China; 2 Department of Cardiology, the First Affiliated Hospital, Guizhou Medical University, 28 Guyi Street, Guiyang, 550000 Guizhou People’s Republic of China; 3 Guangxi Key Laboratory Base of Precision Medicine in Cardio-cerebrovascular Disease Control and Prevention, 6 Shuangyong Road, Nanning, 530021 Guangxi People’s Republic of China; 4 Guangxi Clinical Research Center for Cardio-cerebrovascular Diseases, 6 Shuangyong Road, Nanning, 530021 Guangxi People’s Republic of China; 5 Department of Cardiology, Shaoyang Central Hospital, 36 QianYuan lane, Shaoyang, 422000 Hunan People’s Republic of China; 6 Clinical Laboratory of The Affiliated Cancer Hospital, Guangxi Medical University, 71 Hedi Road, Nanning, 530021 Guangxi People’s Republic of China

**Keywords:** Coronary artery disease, Ischaemic stroke, Gene ontology annotation, Kyoto encyclopedia of genes and genomes (KEGG) pathway, Database for annotation visualization and integrated discovery (DAVID), Protein-protein interaction (PPI) network, RT-qPCR, Unconditional logistic regression

## Abstract

**Background:**

The current research aimed to expound the genes and pathways that are involved in coronary artery disease (CAD) and ischaemic stroke (IS) and the related mechanisms.

**Methods:**

Two array CAD datasets of (GSE66360 and GSE97320) and an array IS dataset (GSE22255) were downloaded. Differentially expressed genes (DEGs) were identified using the limma package. The online tool Database for Annotation, Visualization and Integrated Discovery (DAVID) (version 6.8; david.abcc.ncifcrf.gov) was used to annotate the Kyoto Encyclopedia of Genes and Genomes (KEGG) pathway and Gene Ontology (GO) enrichment analyses of the DEGs. A protein-protein interaction (PPI) network was constructed by Cytoscape software, and then Molecular Complex Detection (MCODE) analysis was used to screen for hub genes. The hub genes were also confirmed by RT-qPCR and unconditional logistic regression analysis in our CAD and IS patients.

**Results:**

A total of 20 common DEGs (all upregulated) were identified between the CAD/IS and control groups. Eleven molecular functions, 3 cellular components, and 49 biological processes were confirmed by GO enrichment analysis, and the 20 common upregulated DEGs were enriched in 21 KEGG pathways. A PPI network including 24 nodes and 68 edges was constructed with the STRING online tool. After MCODE analysis, the top 5 high degree genes, including Jun proto-oncogene (*JUN*, degree = 9), C-X-C motif chemokine ligand 8 (*CXCL8*, degree = 9), tumour necrosis factor (*TNF*, degree = 9), suppressor of cytokine signalling 3 (*SOCS3*, degree = 8) and TNF alpha induced protein 3 (*TNFAIP3*, degree = 8) were noted. RT-qPCR results demonstrated that the expression levels of CXCL8 were increased in IS patients than in normal participants and the expression levels of SOCS3, TNF and TNFAIP were higher in CAD/IS patients than in normal participants. Meanwhile, unconditional logistic regression analysis revealed that the incidence of CAD or IS was positively correlated with the *CXCL8*, *SOCS3*, *TNF* and *TNFAIP3*.

**Conclusions:**

The *CXCL8, TNF, SOCS3* and *TNFAIP3* associated with inflammation may serve as biomarkers for the diagnosis of CAD or IS. The possible mechanisms may involve the Toll-like receptor, TNF, NF-kappa B, cytokine-cytokine receptor interactions and the NOD-like receptor signalling pathways.

## Background

Coronary artery disease (CAD) and ischaemic stroke (IS) are prominent causes of disability, mortality, morbidity, functional deterioration and healthcare expenses and account for approximately 30% of all deaths worldwide [1–4]. Twins and family studies have proven that both CAD and IS are highly heritable [5, 6], and hereditary elements are thought to account for approximately 30–60% of CAD and IS cases [7]. Atherosclerosis is generally regarded as the pathological foundation of CAD [8] and IS [9]. In addition, there is some evidence of several shared genetic characteristics of both diseases [10]. Both diseases are risk factors for one another [11, 12], and they are considered to be therapeutic targets for clinical research and for evaluating the risk of major adverse cardiac events (MACEs). A recent study showed that CAD and IS result from various factors and can be influenced by genomic background, lifestyle, environmental factors and alterations of plasma lipid levels as well as their interactions with each other [13]. To some extent, there is a consensus on the effectiveness of the early prevention of CAD and IS.

As a novel and practical approach for identifying CAD and IS susceptibility genes, a microarray analysis may be helpful for the early diagnosis of CAD and IS [14, 15]. However, the sensitivity and reproducibility of microarray results may be limited [16, 17]. Thus, a comprehensive analysis may be useful to improve the reliability and integrity of the conclusions. Through this method, we can achieve a more accurate approach of identifying susceptibility genes for CAD and IS and further explore their potential biological functions. The Gene Expression Omnibus (GEO, http://www.ncbi.nlm.nih.gov/geo/) [18] is an international public database for next-generation sequence functional genomic datasets and high-throughput microarray data submitted by researchers worldwide. In this study, we downloaded two CAD datasets (GSE66360 and GSE97320) and one IS dataset (GSE22255) to identify differentially expressed genes (DEGs) in patients suffering from CAD or IS and healthy controls. The purpose of the present research was to confirm new biomarkers for the early diagnosis of CAD and IS.

## Materials and methods

### CAD and IS microarray data sets

Two CAD datasets (GSE66360 and GSE97320) and another IS dataset pf IS (GSE22255) were obtained from the GPL570 Affymetrix Human Genome U133 Plus 2.0 array. The GSE22255 dataset included 20 normal samples and 20 IS samples. An integrated analysis of 53 normal samples and 52 CAD samples from the two CAD datasets was performed. The original files in CEL format were transformed into an expression value matrix using the Affy package in R with the RMA method to normalize the expression values and the SVA method to remove batch differences [19]. Then, the bioconductor package was used to transform the probe ID into a gene symbol [20]. When multiple probes corresponded to one common gene, the average value was taken as its expression value.

### Differentially expressed gene (DEG) identification

The DEGs between patients suffering from CAD or IS and healthy participants were identified using the limma package [21]. The threshold values were *P* < 0.05 and |log fold change (FC)| > 1. To visualize the shared DEGs between the CAD datasets and the IS dataset, an online tool (bioinformatics.psb.ugent.be/webtools/Venn) was used to draw a Venn diagram.

### Functional enrichment analysis

The online tool Database for Annotation, Visualization and Integrated Discovery (DAVID) (version 6.8; david.abcc.ncifcrf.gov) was used to annotate the Kyoto Encyclopedia of Genes and Genomes (KEGG) pathway [22] and Gene Ontology (GO) enrichment analyses [23] of the common differentially expressed genes. *P* < 0.05 was defined as the threshold for significant enrichment for KEGG and GO analyses.

### PPI interaction network construction and module analysis

A PPI interaction network of common DEGs was constructed with the Search Tool for the Retrieval of Interacting Genes database (version 11.0; www.string-db.org) [24], and a combined score of > 0.9 was defined as the cut-off value. Cytoscape 3.7.1 (www.cytoscape.org) was applied to visualize the PPI network [25]. Degrees were used to verify the significance of protein nodes in the PPI network. As one of the core components of the PPI network, the network module may have specific biological functions. The Cytoscape software (version 3.61) Molecular Complex Detection (MCODE) plugin was used to identify the most common and largest module clusters with the following parameters: EASE ≤0.05, count ≥2 and MCODE score > 6 [26].

### Sample verification and diagnostic criteria

A total of 420 unrelated participants (202 IS patients and 218 CAD patients) were recruited from the First Affiliated Hospital of Guangxi Medical University from Jan. 1, 2015 to Dec. 31, 2016. CAD was defined as significantly coronary artery stenosis (≥ 50%) in at least anyone of the three main coronary vessels or their main branches (branch diameter ≥ 2 mm) [27]. All patients with IS received a brain magnetic resonance imaging (MRI) scan and strict neurological examination. The diagnostic criteria for IS were derived from the International Classification of Diseases (9th Revision). All subjects with a history of type 1 diabetes, neoplasm, autoimmune disorder, abnormal renal or liver function, haemopathy or thyroid dysfunction were excluded. The patients with CAD had no history of IS, and the patients with IS had no history of CAD.

A total of 203 healthy controls matched by ethnic group (Han Chinese), age, and gender were also recruited. All subjects were healthy, and none of them had a history of CAD, myocardial infarction, IS or type 2 diabetes mellitus (T2DM), as determined by history-taking, questionnaires, or critical clinical examination. All participants were randomly recruited from the Physical Examination Center of the First Affiliated Hospital, Guangxi Medical University in the same period. Before the beginning of the study, all participants signed a written informed consent form. The research proposal was approved by the Ethics Committee of the First Affiliated Hospital, Guangxi Medical University (No: Lunshen-2011-KY-Guoji-001; Mar. 7, 2011).

### Quantitative real-time PCR

RT-qPCR was used to validate the four significantly dysregulated mRNAs identified by the microarray results in the 603 subjects. Total RNA was extracted from peripheral blood mononuclear cells (PBMCs) that were separated from blood samples using TRIzol reagent and reverse transcribed into cDNA using the PrimeScript RT reagent kit (Takara Bio, Japan) according to the manufacturer’s instructions. The resulting cDNA was used as a template for RT-qPCR. Supplementary Table 2 shows the sequences of the specific primers designed by Sangon Biotech (Shanghai, China) and used to detect the 5 hub genes. Quantitative RT-PCR was performed using Taq PCR Master Mix Kit (Takara) on an ABI Prism 7500 sequence-detection system (Applied Biosystems, USA) using RT Reaction Mix in a total volume of 20 μL with conditions of 95 °C pre-denaturation for 30 s, 95 °C for 30 s, and 60 °C for 30 s for 40 cycles.

### Diagnostic criteria

In our Clinical Science Experiment Center, 0.56–1.70 mmol/L serum triglyceride (TG), 3.10–5.17 mmol/L total cholesterol (TC), 0.80–1.05 g/L apolipoprotein (Apo) B, 2.70–3.10 mmol/L low-density lipoprotein cholesterol (LDL-C), 1.20–1.60 g/L ApoA1, 1.16–1.42 mmol/L high-density lipoprotein cholesterol (HDL-C) and a ApoA1/ApoB ratio of 1.00–2.50 were defined as normal values. The diagnostic criteria of hyperlipidaemia [28], hypertension [29], obesity, normal weight, and overweight [30] were referred to in previous studies. Participants who had been previously diagnosed with diabetes and participants with 2-h postprandial plasma glucose ≥11.1 mmol/L or fasting plasma glucose ≥7.0 mmol/L were defined as diabetic patients [31].

### Statistical analyses

All data were x (Version 22.0). The values are presented as the mean ± SD. The chi-square test was used to calculate the differences in the rates between patients and controls. Independent samples *t* test was used to analyse differences in general characteristics between patients and controls. Unconditional logistic regression was used to evaluate the relationship between genes and clinical variables and the incidence of CAD or IS. The pheatmap and ggplot2 packages (https://cran.r-project.org/) were used to draw the volcano plot and heat map.

## Results

### Identification of DEGs in GSE97320, GSE66360 and GSE22255

**Fig. 1:**
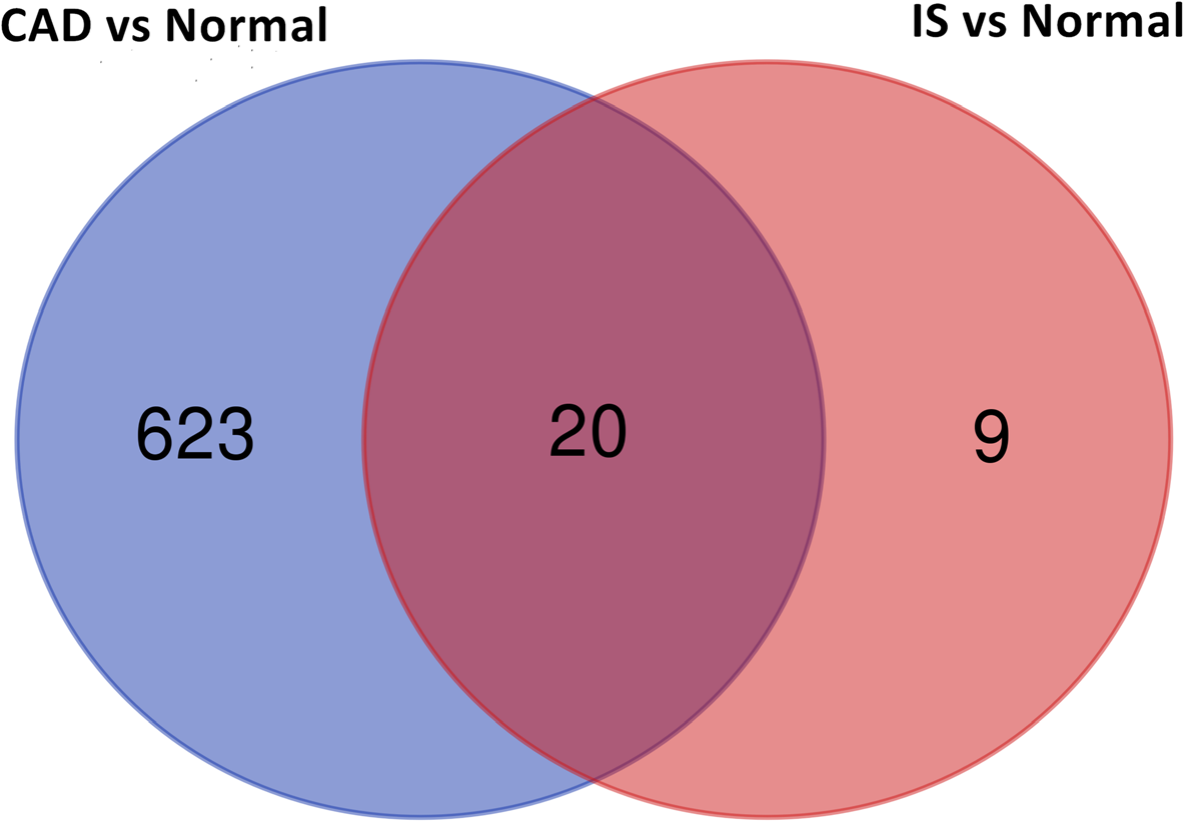
Venn map showing the intersection of DEGs between CAD vs normal and IS vs normal

**Fig. 2:**
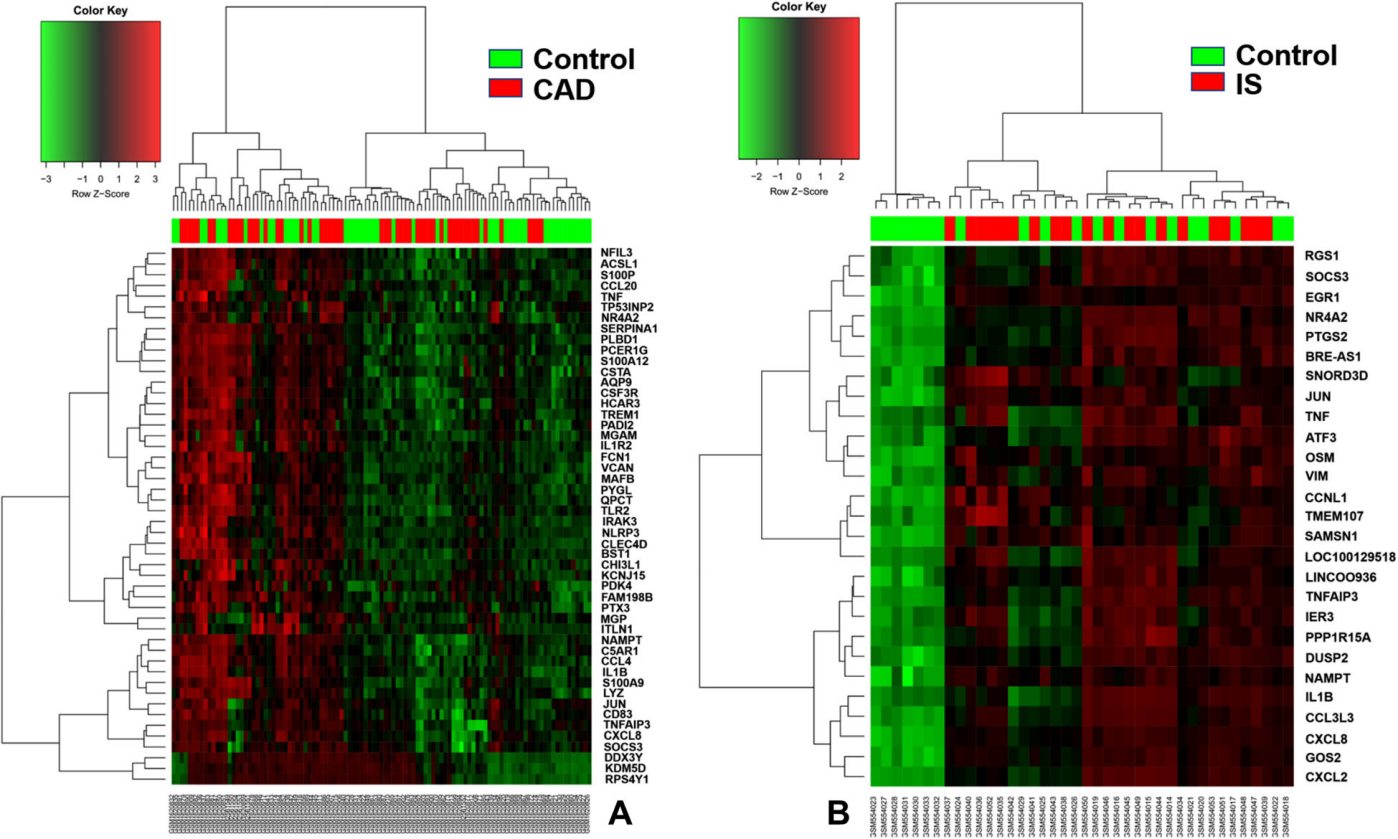
Cluster heat maps of DEGs. Red represents CAD/IS group and green represents control group. **a**: Top 50 up-regulated DEGs between CAD and control; **b**: Top 27 up-regulated and 2 down- regulated DEGs between IS and control

**Fig. 3:**
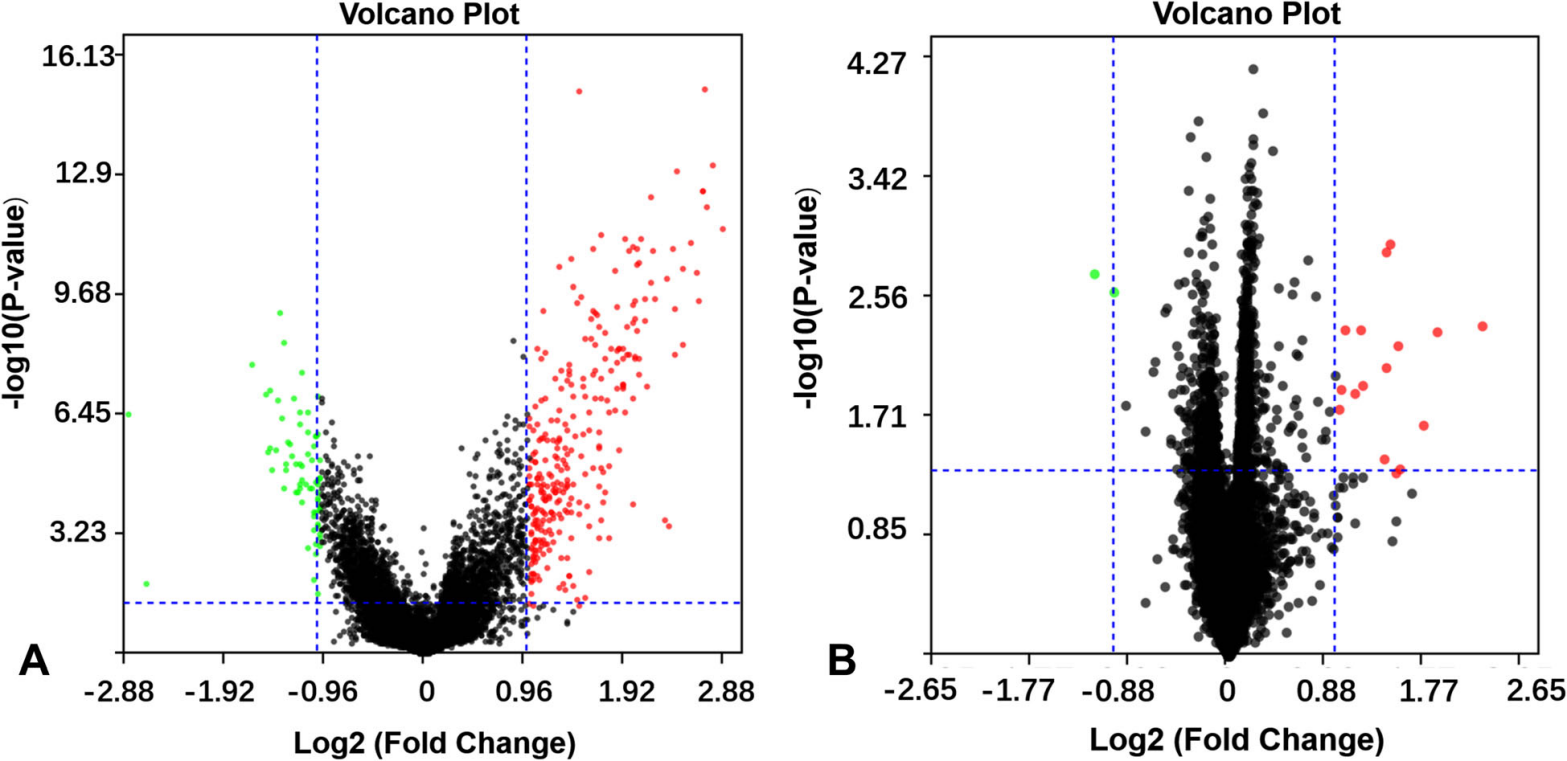
Volcano plots of DEGs. Up-regulated genes are marked with red dots, and down-regulated genes are marked with green dots. **a**: CAD vs. control; **b**: IS vs. control

After data normalization and removed of batch differences, a total of 643 genes, including 178 downregulated genes and 465 upregulated genes, were defined as DEGs between the patients with CAD and healthy controls according to the following criteria: |logFC| > 1 and *P* < 0.05. A total of 29 DEGs, including 2 downregulated genes and 27 upregulated genes were identified between IS patients and healthy controls, and 20 common upregulated DEGs between the CAD patients and controls and between IS patients and controls were identified (Table 1 and Fig. 1). Analysis of heatmap clustering and the volcano plot showed that the identified DEGs can easily distinguish patients with CAD or IS from healthy controls (Figs. 2 and 3)**.**

**Table 1:** All of 20 common upregulated differentially expressed genes between the groups of CAD or IS and control

Upregulated Gene	CAD vs. control		IS vs. control	
	Log fold change	*P*-value	Log fold change	*P*-value
JUN	1.53	1.18E-06	1.53	0.0009
ATF3	1.45	6.13E-12	1.07	0.0306
BRE-AS1	1.04	8.94E-07	1.41	0.0387
CCNL1	1.72	5.53E-09	1.08	0.0018
CXCL2	1.91	6.40E-08	2.27	0.0207
CXCL8	1.50	0.0001	2.65	0.0031
EGR1	1.58	1.66E-05	1.43	0.0086
G0S2	1.35	2.65E-06	2.29	0.0043
IER3	1.94	1.35E-09	1.18	0.0045
IL1B	2.68	3.30E-13	1.55	0.0459
NAMPT	2.03	1.09E-08	1.30	0.0076
NR4A2	2.78	6.33E-14	1.53	0.045
OSM	1.53	3.29E-08	1.17	0.0068
PPP1R15A	1.93	6.47E-12	1.15	0.0132
PTGS2	1.56	9.92E-05	1.76	0.0222
RGS1	1.58	1.30E-06	1.35	0.0259
SAMSN1	1.41	0.0001	1.22	0.0049
SOCS3	1.37	7.20E-07	1.05	0.0291
TNF	1.21	1.45E-05	1.52	0.0059
TNFAIP3	1.34	4.07E-05	1.02	0.0190

### KEGG pathway and GO functional enrichment analysis

**Fig. 4:**
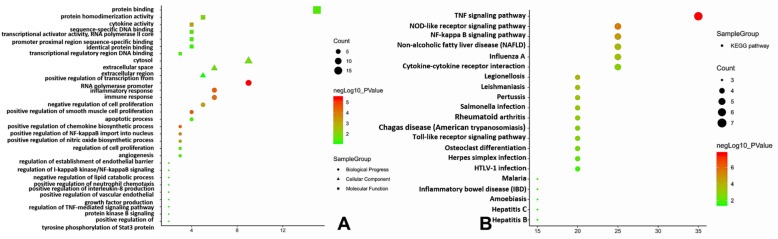
Functional annotation for DEGs. **a** GO enrichment analysis of DEGs; **b** KEGG pathways analysis of DEGs

The online tool DAVID was used to predict the potential biological functions of the DEGs. A total of 21 KEGG pathways, including the Toll-like receptor signalling pathway (*TNF, JUN, CXCL8, and IL1B*); the NF-kappa B signalling pathway (*TNF, CXCL8, IL1B, and TNFAIP3*); the TNF signalling pathway (*TNF, SOCS3, JUN, IL1B,* and *TNFAIP3*), 11 molecular functions, 3 cellular components, and 49 biological processes were enriched in the present study, and GO:0006915~apoptotic process (*IL1B* and *TNFAIP3*), GO:0042346~positive regulation of NF-kB import into nucleus (*TNF* and *IL1B*), GO:0045429~positive regulation of nitric oxide biosynthetic process (*TNF* and *IL1B*), GO:0006954~inflammatory response (*TNF, CXCL8, IL1B,* and *TNFAIP3*), GO:0050995~negative regulation of lipid catabolic process (*TNF* and *IL1B*), GO:0034116~positive regulation of heterotypic cell-cell adhesion (*TNF* and *IL1B*), GO:0048661~positive regulation of smooth muscle cell proliferation (*TNF* and *JUN*), GO:0010803~regulation of tumour necrosis factor-mediated signalling pathway (*TNF* and *TNFAIP3*), GO:0001525~angiogenesis (*JUN* and *CXCL8*) and GO:0043122~regulation of I-kappaB kinase/NF-kB signalling (*TNF* and *IL1B*) were selected for further analysis, as presented in Fig. 4. More detailed information is presented in Supplementary Table 1.

### PPI network construction and module analysis for DEGs

**Fig. 5:**
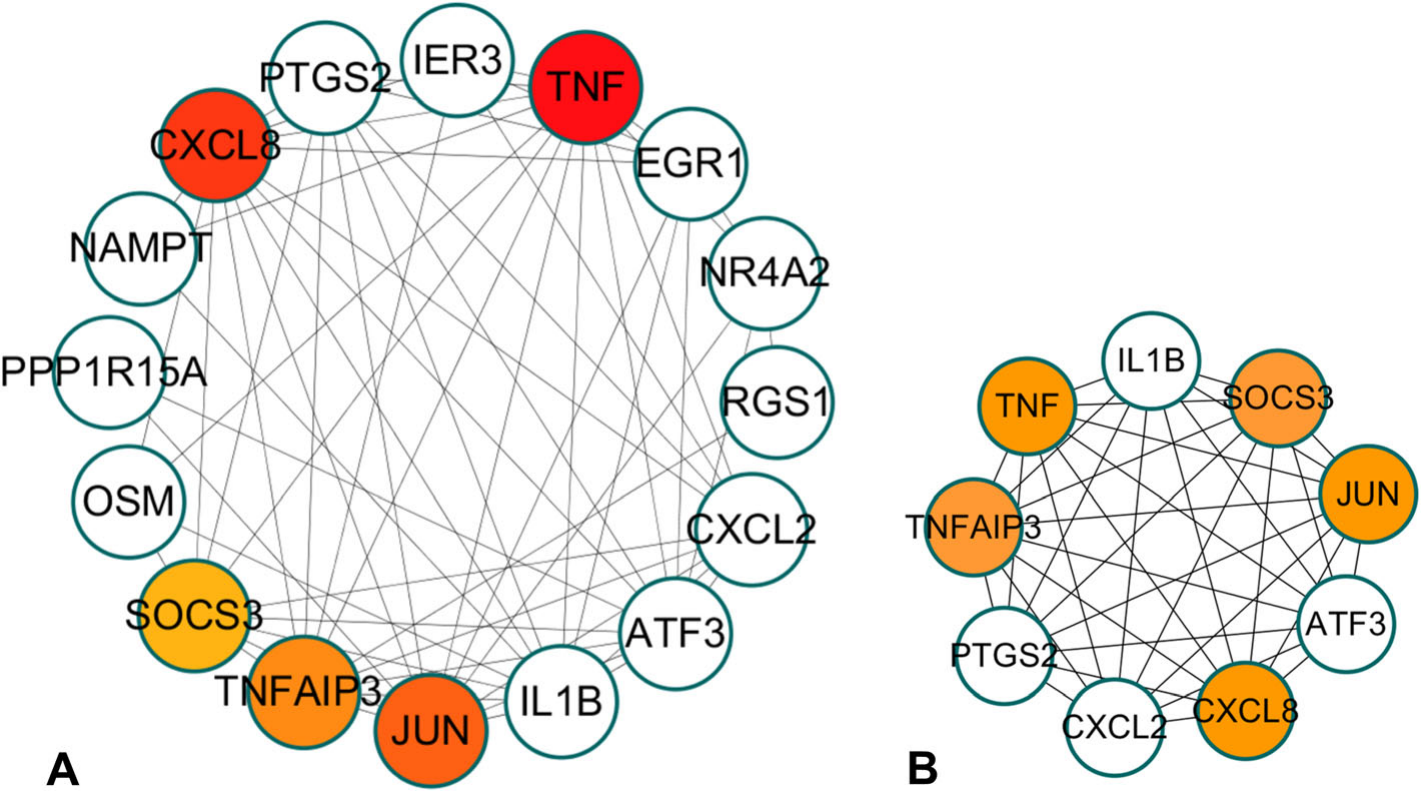
PPI network construction and identification of hub genes. **a** PPI network of the selected DEGs. The edge shows the interaction between two genes. Significant modules identified from the PPI network using the MCODE with a score > 6.0. **b** Moldule-1 with MCODE = 9

A PPI network including 24 nodes and 68 edges was constructed with the STRING online tool. After MCODE analysis, the top 5 high degree genes, including C-X-C motif chemokine ligand 8 (*CXCL8*, degree = 9), Jun proto-oncogene (*JUN*, degree = 9), tumour necrosis factor (*TNF*, degree = 9), suppressor of cytokine signalling 3 (*SOCS3*, degree = 8), and TNF alpha induced protein 3 (*TNFAIP3*, degree = 8), were identified in the present study (Fig. 5).

### Validation by RT-qPCR

**Fig. 6:**
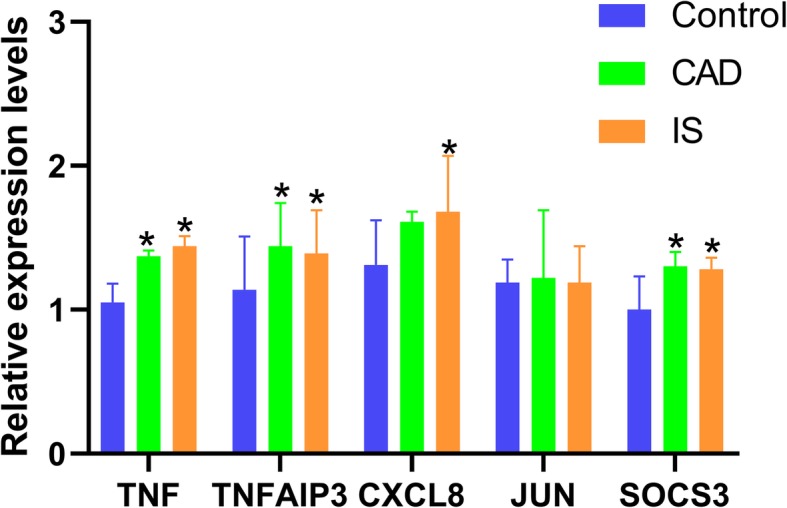
Relative expression levels of five hub genes identified from the microarray data were verified by RT-qPCR. ^*^P < 0.05

The RT-qPCR results revealed that the expression levels of CXCL8 were increased in IS patients than in normal participants and the expression levels of *SOCS3*, *TNF* and *TNFAIP3* genes were higher in CAD/IS patients than in normal participants. Meanwhile, there was no difference in the expression of *JUN* between CAD/IS patients and the control group. The RT-qPCR results in our study were in accordance with the results of the microarray analysis (Fig. 6). The primer sequences for the abovementioned genes are shown in Supplementary Table 2**.**

### Biochemical characteristics and unconditional logistic regression analysis

As mentioned in Table 2, the female to male ratio, age, serum ApoB levels, the proportion of drinkers, height and diastolic blood pressure were similar between the controls and patients. The proportion of smokers, weight, systolic blood pressure, glucose, body mass index (BMI), pulse pressure, and serum LDL-C, TG and TC levels were significantly lower and serum ApoA1, HDL-C levels and the ApoA1/ApoB ratio were significantly higher in controls than in both CAD and IS patients.

**Table 2:** Comparison of demographic, lifestyle characteristics and serum lipid levels of the participants

Characteristic	Control	Case		*P* vs. *controls*	
	(*n* = 203)	CAD (*n* = 218)	Is (*n* = 202)	*P*_CAD_	*P*_IS_
Male/female	149/54	160/58	146/56	0.936	0.670
Age (years)	61.61 ± 11.95	62.32 ± 10.53	62.73 ± 12.37	0.270	0.111
Height (cm)	164.61 ± 7.37	165.18 ± 7.03	163.93 ± 7.33	0.419	0.352
Weight (kg)	53.75 ± 9.10	65.94 ± 10.58	62.94 ± 11.13	0.000	0.000
BMI (kg/m^2^)	19.94 ± 3.76	24.11 ± 3.21	23.34 ± 3.42	0.000	0.000
Smoking, *n* %	67(33.0)	98 (45.0)	88(43.6)	0.013	0.032
Alcohol, *n* %)	54(26.6)	60(27.5)	57(28.2)	0.913	0.739
SBP (mmHg)	130.00 ± 18.91	136.70 ± 22.63	147.78 ± 21.02	0.001	0.000
DBP (mmHg)	80.78 ± 11.45	79.30 ± 13.19	82.78 ± 11.61	0.219	0.082
PP (mmHg)	49.22 ± 14.39	57.40 ± 19.97	65.00 ± 17.15	0.000	0.000
Glu (mmol/L)	6.08 ± 1.80	6.46 ± 1.87	6.44 ± 1.52	0.034	0.030
TC (mmol/L)	4.43 ± 1.00	4.79 ± 1.35	4.70 ± 1.05	0.003	0.010
TG (mmol/L)	1.35 ± 1.20	1.76 ± 1.12	1.63 ± 1.60	0.000	0.049
HDL-C (mmol/L)	1.85 ± 0.47	1.15 ± 0.32	1.25 ± 0.37	0.000	0.000
LDL-C (mmol/L)	2.76 ± 0.98	3.03 ± 1.09	2.95 ± 0.81	0.008	0.034
ApoA1 (g/L)	1.41 ± 0.33	1.01 ± 0.31	1.02 ± 0.22	0.000	0.000
ApoB (g/L)	0.93 ± 0.20	0.95 ± 0.27	0.89 ± 0.26	0.378	0.084
ApoA1/ApoB	1.58 ± 0.47	1.15 ± 0.50	1.28 ± 0.49	0.000	0.000

*SBP* Systolic blood pressure, *DBP* Diastolic blood pressure, *PP* Pulse pressure, *Glu* Glucose, *HDL-C* high-density lipoprotein cholesterol, *LDL-C* low-density lipoprotein cholesterol, *Apo* Apolipoprotein, *TC* Total cholesterol*, TG* Triglyceride

^a^ Continuous data were presented as means ± SD and determined by two side *t*-test

^b^ A chi-square analysis was used to evaluate the difference of the rate between the groups

**Fig. 7:**
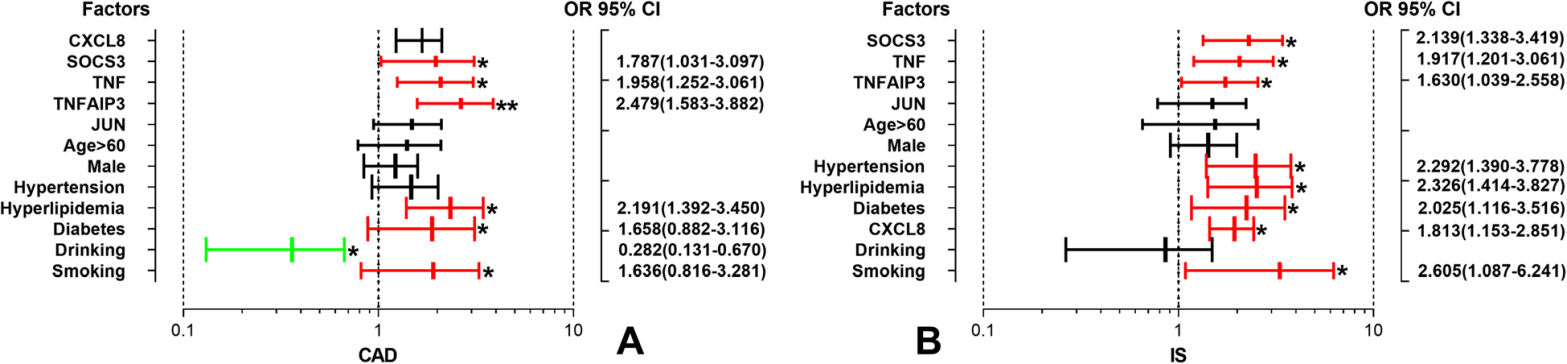
The relative risk factors for CAD and IS *CAD* coronary artery disease; *IS* ischemic stroke. ^*^P < 0.05. ^**^P < 0.01

Unconditional logistic regression analysis revealed that the overexpression of *CXCL8*, *SOCS3*, *TNF* and *TNFAIP3*, hyperlipidaemia, smoking and diabetes were considered independent risk factors for the incidence of CAD or IS; the incidence of IS was also positively correlated with hypertension, and the incidence of CAD was negatively correlated with alcohol consumption (Fig. 7).

## Discussion

Currently, the diagnosis of CAD is based on ischaemia-related symptoms, detailed physical examination, electrocardiogram changes, elevated biomarkers of myocardial injury and coronary angiography [32, 33]. Meanwhile, the diagnosis of ischaemic stroke (IS) is also based on the patient’s symptoms, signs, strict neurological examination and MRI scans [34]. However, the early diagnosis of CAD and IS is still limited. As a novel and practical approach for identifying CAD and IS susceptibility genes, microarray analysis may be helpful for the early diagnosis of CAD and IS [14, 15]. However, the sensitivity and reproducibility of microarray results may be limited [16, 17]. Thus, it is important for us to identify several new biomarkers for the early diagnosis of CAD and IS through the integrated analysis of different datasets. Therefore, in the present research, we integrated and analysed two different CAD datasets and an IS dataset, identified 20 common DEGs to further and analysed their KEGG pathways, GO functional enrichment, and PPI networks and modules to define five significantly DEGs (*CXCL8*, *TNF*, *SOCS3*, *TNFAIP3*, and *JUN*). However, when we verified the above results in our experiment, we found that the expression of *CXCL8*, *TNF*, *SOCS3*, and *TNFAIP3* was higher in patients with CAD or IS than in healthy controls and that there was no significant difference in the expression of *JUN* between CAD or IS patients and the control group.

A recent study showed that CAD and IS result from various factors and can be influenced by genomic background, lifestyle, environmental factors, alterations in plasma lipid levels and the interactions of these factors [13]. Atherosclerosis is generally regarded as the pathological foundation of CAD [8] and IS [9]. Actually, atherosclerosis is not only a lipid-driven disease, but also a type of chronic inflammatory process involving numerous inflammatory cells and mediators [35]. The toll-like receptor signalling pathway plays a crucial role in adaptive and innate immune responses and represents an important medium between inflammation and atherosclerosis [36]. Toll-like receptors are the most characteristic pattern recognition receptors in the innate immune system and are expressed in all types of leukocytes, such as B, T and DC lymphocytes and macrophages/monocytes. The involvement of Toll-like receptors in immune and inflammatory responses may play a crucial role in various aspects of the formation and development of atherosclerotic lesions, and this effect may be related to multiple biological processes, including foam cell formation, the induction of leukocyte recruitment, lipid uptake and proinflammatory cytokine release, which are all facilitated by Toll-like receptors [37]. In the present study, enrichment analysis of KEGG pathways suggested that the *TNF*, *CXCL8* and *IL1B* genes may be involved in the Toll-like receptor signalling pathway. Thus, we speculated that these genes might exert their biological functions through the Toll-like receptor signalling pathway.

The TNF signalling pathway plays a crucial role in inflammatory and autoimmune diseases. TNF-α is one of the most important members of the TNF superfamily is mainly secreted by macrophages and participates in the regulation of a wide spectrum of biological processes, including cell proliferation, lipid metabolism, apoptosis and differentiation. All of the above biological processes can lead to chronic immunoinflammatory lesions that eventually result in atherosclerosis [38]. Previous studies have proven that the NF-kappa B (NF-kB) signalling pathway plays a key role in the inflammatory reaction, leading to the transcription of genes involved in endothelial inflammation and injury. TNF-α is a major inflammatory cytokine involved in activating the NF-kB signalling pathway to induce the production of more inflammation mediators and reactive oxygen species [39, 40]. At the same time, numerous scientific studies have shown that, in atherogenesis, increased levels of IL-1β and TNF-α, as the two leading mediators of the inflammatory response, in result from increased transcriptional activity of the NF-kB gene [41, 42]. A recent compelling study showed that quercetin may play an anti-inflammatory role in the treatment of stable coronary heart disease by reducing the transcriptional activity of the NF-kB gene [43]. Similar studies have also shown that NF-kB acts as a key regulator of various genes involved in inflammation and cell survival and is activated after cerebral ischaemia in microglia, neurons, astrocytes and infiltrating inflammatory cells [44]. These results show that the TNF and NF-kB signalling pathways may be involved in the development of CAD and IS. In the present study, enrichment analysis of KEGG pathways indicated that the *TNF*, *SOCS3*, *JUN*, and *TNFAIP3* genes may be involved in the TNF signalling pathway and that *TNF*, *CXCL8* and *TNFAIP3* may be involved in the NF-kB signalling pathway. Meanwhile, cytokine-cytokine receptor interactions (TNF, CXCL8, and IL1B) and the NOD-like receptor signalling pathway (TNF, CXCL8, IL1B, TNFAIP3) were also identified in our study.

In addition, several main biological processes that may be involved in chronic inflammatory lesions that eventually result in atherosclerosis were identified by GO functional enrichment analysis of DEGs, such as GO:0006915~apoptotic process (*TNFAIP3*), GO:0042346~positive regulation of NF-kB import into nucleus (*TNF*), GO:0045429~positive regulation of nitric oxide biosynthetic process (*TNF*), GO:0006954~inflammatory response (*TNF*, *CXCL8* and *TNFAIP3*), GO:0050995~negative regulation of lipid catabolic process (*TNF*), GO:0048661~positive regulation of smooth muscle cell proliferation (*TNF*), GO:0010803~regulation of tumour necrosis factor-mediated signalling pathway (*TNF* and *TNFAIP3*), GO:0001525~angiogenesis (*CXCL8*), GO:0042517~positive regulation of tyrosine phosphorylation of Stat3 protein (*SOCS3*) and GO:0043122~regulation of I-kappaB kinase/NF-kB signalling (*TNF*). This information about the relevant pathways is not novel; however, the analytical approach is different from that of previous studies. Thus, the combination of previous and current research results revealed that *TNF*, *CXCL8*, *SOCS3* and *TNFAIP3* may be involved in chronic inflammatory lesions that eventually result in atherosclerosis, CAD or IS. Furthermore, RT-qPCR and unconditional logistic regression also validated the above results in our CAD and IS patients. We obtained results that were consistent with those of the microarray analysis, which might increase the credibility of the conclusions.

## Conclusions

Two microarray CAD datasets and an IS dataset were integrated and analysed in the present study. Five hub genes (*SOCS3*, *JUN*, *TNF*, *CXCL8*, and *TNFAIP3*) were identified following GO functional enrichment analysis, KEGG pathway enrichment analysis, PPI network construction and MCODE analysis, but only four genes (*SOCS3*, *TNF*, *CXCL8*, and *TNFAIP3*) were verified by RT-qPCR in our CAD or IS patients. The CXCL8, TNF, SOCS3, TNFAIP3 genes, which are associated with inflammation, may serve as biomarkers for the diagnosis of CAD or IS. The mechanism may involve the TNF signalling pathway, the Toll-like receptor signalling pathway, the NF-kappa B signalling pathway, cytokine-cytokine receptor interactions and the NOD-like receptor signalling pathway.

## Supplementary information

**Additional file 1 **: **Table S1** KEGG pathways and GO function enrichment analyses of twenty common upregulated-DEGs. **Table S2** PCR primers for quantitative real-time PCR

## Data Availability

The datasets used and/or analysed during the current study are available from the corresponding author on reasonable request.

## Abbreviations

Apo: Apolipoprotein
BMI: Body mass index
CAD: Coronary artery disease
CXCL8: C-X-C motif chemokine ligand 8
DAVID: Database for Annotation, Visualization and Integrated Discovery
DEGs: Differentially expressed genes
GEO: Gene Expression Omnibus
GO: Gene Ontology
HDL-C: High-density lipoprotein cholesterol
IS: Ischemic stroke
JUN: Jun proto-oncogene
KEGG: Kyoto Encyclopedia of Genes and genomes
LDL-C: Low-density lipoprotein cholesterol
logFC: Log fold change
MACEs: Major adverse cardiac events
MCODE: Molecular Complex Detection
MRI: Magnetic resonance imaging
NF-kB: NF-kappa B
PPI: Protein-protein interaction
RT-PCR: SOCS3: Suppressor of cytokine signaling 3
T2DM: Type 2 diabetes mellitus
TC: Total cholesterol
TG: Triglyceride
TNF: Tumor necrosis factor
TNFAIP3: TNF alpha induced protein 3
