# Supplementary material for: Genes associated with inflammation may serve as biomarkers for the diagnosis of coronary artery disease and ischaemic stroke
Source: Lipids Health Dis. 2020 Mar 12;19:37. doi: 10.1186/s12944-020-01217-7 (PMC7066794; doi:10.1186/s12944-020-01217-7)
Supplement: Supplementary file 1 — Additional file 1 : Table S1 KEGG pathways and GO function enrichment analyses of twenty common upregulated-DEGs. Table S2 PCR primers for quantitative real-time PCR [file 12944_2020_1217_MOESM1_ESM.docx]

**Supplementary Table 1** PCR primers for quantitative real-time PCR

| **Gene** | **Forward primer** | **Reverse primer** |
| --- | --- | --- |
| CXCL8 | AGGTGCAGTTTTGCCAAGGA | CAACCCTCTGCACCCAGTTT |
| TNFAIP3 | GCCAAGAGAGATCACACCCC | TTCGTTTTCAGCGCCACAAG |
| TNF | CACAGTGAAGTGCTGGCAAC | ACATTGGGTCCCCCAGGATA |
| JUN | GTCCGAGAGCGGACCTTATG | CTTTTTCGGCACTTGGAGGC |
| SOCS3 | GGGGAGTACCACCTGAGTCT | TGTGGTTGCTATCGTCCCAC |
| GAPDH | AGAGAGAGGCCCTCAGTTGCT | TTGTGAGGGAGATGCTCAGTGT |

**Supplementary Table 2** KEGG pathways and GO function enrichment analyses of twenty common upregulated-DEGs

| **Term** | ***P*-value** | **Gene** |
| --- | --- | --- |
| **Biological process** |  |  |
| GO:0051781~positive regulation of cell division | 0.049 | OSM, IL1B |
| GO:0032729~positive regulation of interferon-gamma production | 0.048 | TNF, IL1B |
| GO:0032755~positive regulation of interleukin-6 production | 0.047 | TNF, IL1B |
| GO:0007566~embryo implantation | 0.044 | PTGS2, IL1B |
| GO:0097191~extrinsic apoptotic signaling pathway | 0.044 | TNF, G0S2 |
| GO:0042517~positive regulation of tyrosine phosphorylation of Stat3 protein | 0.040 | OSM, SOCS3 |
| GO:0000122~negative regulation of transcription from RNA polymerase II promoter | 0.040 | EGR1, TNF, ATF3, NR4A2 |
| GO:2001240~negative regulation of extrinsic apoptotic signaling pathway in absence of ligand | 0.039 | TNF, IL1B |
| GO:0045597~positive regulation of cell differentiation | 0.039 | SOCS3, JUN |
| GO:0043491~protein kinase B signaling | 0.035 | TNF, IL1B |
| GO:0031663~lipopolysaccharide-mediated signaling pathway | 0.034 | TNF, IL1B |
| GO:0007165~signal transduction | 0.032 | NAMPT, RGS1, NR4A2, CXCL8, IL1B |
| GO:0010803~regulation of tumor necrosis factor-mediated signaling pathway | 0.032 | TNF, TNFAIP3 |
| GO:0050766~positive regulation of phagocytosis | 0.031 | TNF, IL1B |
| GO:0046627~negative regulation of insulin receptor signaling pathway | 0.031 | SOCS3, IL1B |
| GO:0032715~negative regulation of interleukin-6 production | 0.030 | TNF, TNFAIP3 |
| GO:0010575~positive regulation of vascular endothelial growth factor production | 0.029 | PTGS2, IL1B |
| GO:0032757~positive regulation of interleukin-8 production | 0.028 | TNF, IL1B |
| GO:0001525~angiogenesis | 0.023 | PTGS2, JUN, CXCL8 |
| GO:0090023~positive regulation of neutrophil chemotaxis | 0.023 | CXCL2, CXCL8 |
| GO:0006915~apoptotic process | 0.021 | IER3, IL1B, TNFAIP3, PPP1R15A |
| GO:0048566~embryonic digestive tract development | 0.017 | TNF, CXCL8 |
| GO:0050995~negative regulation of lipid catabolic process | 0.017 | TNF, IL1B |
| GO:0045893~positive regulation of transcription, DNA-templated | 0.017 | EGR1, TNF, JUN, IL1B |
| GO:0042127~regulation of cell proliferation | 0.016 | TNF, JUN, CXCL2 |
| GO:0051044~positive regulation of membrane protein ectodomain proteolysis | 0.016 | TNF, IL1B |
| GO:0043122~regulation of I-kappaB kinase/NF-kappaB signaling | 0.016 | TNF, IL1B |
| GO:0032496~response to lipopolysaccharide | 0.013 | PTGS2, JUN, CXCL2 |
| GO:0036499~PERK-mediated unfolded protein response | 0.013 | ATF3, CXCL8 |
| GO:0034116~positive regulation of heterotypic cell-cell adhesion | 0.012 | TNF, IL1B |
| GO:0006769~nicotinamide metabolic process | 0.007 | NAMPT, PTGS2 |
| GO:0050869~negative regulation of B cell activation | 0.007 | TNFAIP3, SAMSN1 |
| GO:1903140~regulation of establishment of endothelial barrier | 0.007 | TNF, IL1B |
| GO:0071222~cellular response to lipopolysaccharide | 0.006 | TNF, CXCL8, TNFAIP3 |
| GO:1990441~negative regulation of transcription from RNA polymerase II promoter in response to endoplasmic reticulum stress | 0.005 | JUN, PPP1R15A |
| GO:0030730~sequestering of triglyceride | 0.004 | TNF, IL1B |
| GO:0060559~positive regulation of calcidiol 1-monooxygenase activity | 0.003 | TNF, IL1B |
| GO:0033138~positive regulation of peptidyl-serine phosphorylation | 0.002 | OSM, TNF, PPP1R15A |
| GO:0045429~positive regulation of nitric oxide biosynthetic process | 9.55E-04 | TNF, PTGS2, IL1B |
| GO:0008285~negative regulation of cell proliferation | 7.17E-04 | OSM, PTGS2, JUN, CXCL8, IL1B |
| GO:0042346~positive regulation of NF-kappaB import into nucleus | 2.25E-04 | TNF, PTGS2, IL1B |
| GO:0006955~immune response | 6.33E-05 | OSM, TNF, RGS1, CXCL2, CXCL8, IL1B |
| GO:0045080~positive regulation of chemokine biosynthetic process | 4.86E-05 | EGR1, TNF, IL1B |
| GO:0002237~response to molecule of bacterial origin | 3.89E-05 | CXCL2, CXCL8, TNFAIP3 |
| GO:0006954~inflammatory response | 3.84E-05 | TNF, PTGS2, CXCL2, CXCL8, IL1B, TNFAIP3 |
| GO:0048661~positive regulation of smooth muscle cell proliferation | 3.41E-05 | NAMPT, TNF, PTGS2, JUN |
| GO:0031622~positive regulation of fever generation | 1.08E-05 | TNF, PTGS2, IL1B |
| GO:0045944~positive regulation of transcription from RNA polymerase II promoter | 3.41E-06 | OSM, EGR1, NAMPT, TNF, ATF3, JUN, NR4A2, CCNL1, IL1B |
| **Molecular function** |  |  |
| GO:0005125~cytokine activity | 8.10E-04 | OSM, NAMPT, TNF, IL1B |
| GO:0042803~protein homodimerization activity | 0.006 | NAMPT, ATF3, PTGS2, JUN, NR4A2 |
| GO:0043565~sequence-specific DNA binding | 0.017 | EGR1, ATF3, JUN, NR4A2 |
| GO:0044212~transcription regulatory region DNA binding | 0.021 | TNF, ATF3, JUN |
| GO:0005515~protein binding | 0.023 | EGR1, NAMPT, IER3, TNF, PTGS2, SOCS3, CXCL2, CCNL1, NR4A2, CXCL8, ATF3, JUN, G0S2, TNFAIP3, PPP1R15A |
| GO:0000982~transcription factor activity, RNA polymerase II core promoter proximal region sequence-specific binding | 0.024 | ATF3, JUN |
| GO:0001077~transcriptional activator activity, RNA polymerase II core promoter proximal region sequence-specific binding | 0.026 | EGR1, JUN, NR4A2 |
| GO:0042802~identical protein binding | 0.043 | TNF, ATF3, JUN, TNFAIP3 |
| GO:0008009~chemokine activity | 0.051 | CXCL2, CXCL8 |
| GO:0000976~transcription regulatory region sequence-specific DNA binding | 0.060 | EGR1, ATF3 |
| GO:0046982~protein heterodimerization activity | 0.087 | ATF3, JUN, NR4A2 |
| **Cellular component** |  |  |
| GO:0005615~extracellular space | 0.008 | OSM, NAMPT, TNF, CXCL2, CXCL8, IL1B |
| GO:0005829~cytosol | 0.009 | IER3, NAMPT, RGS1, SOCS3, JUN, IL1B, TNFAIP3, SAMSN1, PPP1R15A |
| GO:0005576~extracellular region | 0.068 | OSM, TNF, CXCL2, CXCL8, IL1B |
| **KEGG pathways** |  |  |
| hsa04668: TNF signaling pathway | 1.93E-08 | TNF, PTGS2, SOCS3, JUN, CXCL2, IL1B, TNFAIP3 |
| hsa04621: NOD-like receptor signaling pathway | 2.67E-06 | TNF, CXCL2, CXCL8, IL1B, TNFAIP3 |
| hsa04064: NF-kappa B signaling pathway | 1.56E-05 | TNF, PTGS2, CXCL8, IL1B, TNFAIP3 |
| hsa05134: Legionellosis | 1.24E-04 | TNF, CXCL2, CXCL8, IL1B |
| hsa04932: Non-alcoholic fatty liver disease (NAFLD) | 1.37E-04 | TNF, SOCS3, JUN, CXCL8, IL1B |
| hsa05164: Influenza A | 2.37E-04 | TNF, SOCS3, JUN, CXCL8, IL1B |
| hsa05140: Leishmaniasis | 2.80E-04 | TNF, PTGS2, JUN, IL1B |
| hsa05133: Pertussis | 3.29E-04 | TNF, JUN, CXCL8, IL1B |
| hsa05132: Salmonella infection | 4.44E-04 | JUN, CXCL2, CXCL8, IL1B |
| hsa05323: Rheumatoid arthritis | 5.27E-04 | TNF, JUN, CXCL8, IL1B |
| hsa04060: Cytokine-cytokine receptor interaction | 8.44E-04 | OSM, TNF, CXCL2, CXCL8, IL1B |
| hsa05142: Chagas disease (American trypanosomiasis) | 8.60E-04 | TNF, JUN, CXCL8, IL1B |
| hsa04620: Toll-like receptor signaling pathway | 9.09E-04 | TNF, JUN, CXCL8, IL1B |
| hsa04380: Osteoclast differentiation | 0.002 | TNF, SOCS3, JUN, IL1B |
| hsa05144: Malaria | 0.004 | TNF, CXCL8, IL1B |
| hsa05168: Herpes simplex infection | 0.004 | TNF, SOCS3, JUN, IL1B |
| hsa05321: Inflammatory bowel disease (IBD) | 0.006 | TNF, JUN, IL1B |
| hsa05166: HTLV-I infection | 0.011 | EGR1, TNF, ATF3, JUN |
| hsa05146: Amoebiasis | 0.016 | TNF, CXCL8, IL1B |
| hsa05160: Hepatitis C | 0.025 | TNF, SOCS3, CXCL8 |
| hsa05161: Hepatitis B | 0.030 | TNF, JUN, CXCL8 |
